# Fabrication of a Biocompatible Nanoantimicrobial Suture for Rapid Wound Healing after Surgery

**DOI:** 10.1021/acsomega.3c09484

**Published:** 2024-05-13

**Authors:** Tuba Baygar, Aysel Ugur, Inci Rana Karaca, Yeliz Kilinc, Sibel Elif Gultekin, Nurdan Sarac

**Affiliations:** † Material Research Laboratory, Research Laboratories Center, Mugla Sitki Kocman University, Mugla 48000, Turkey; ‡ Faculty of Dentistry, Department of Basic Sciences, Section of Medical Microbiology, Gazi University, Ankara 06500, Turkey; § Faculty of Dentistry, Department of Oral and Maxillofacial Surgery, Gazi University, Ankara 06500, Turkey; ∥ Faculty of Dentistry, Department of Basic Sciences, Department of Oral Pathology, Gazi University, Ankara 06500, Turkey; ⊥ Department of Biology, Faculty of Science, Mugla Sitki Kocman University, Mugla 48000, Turkey

## Abstract

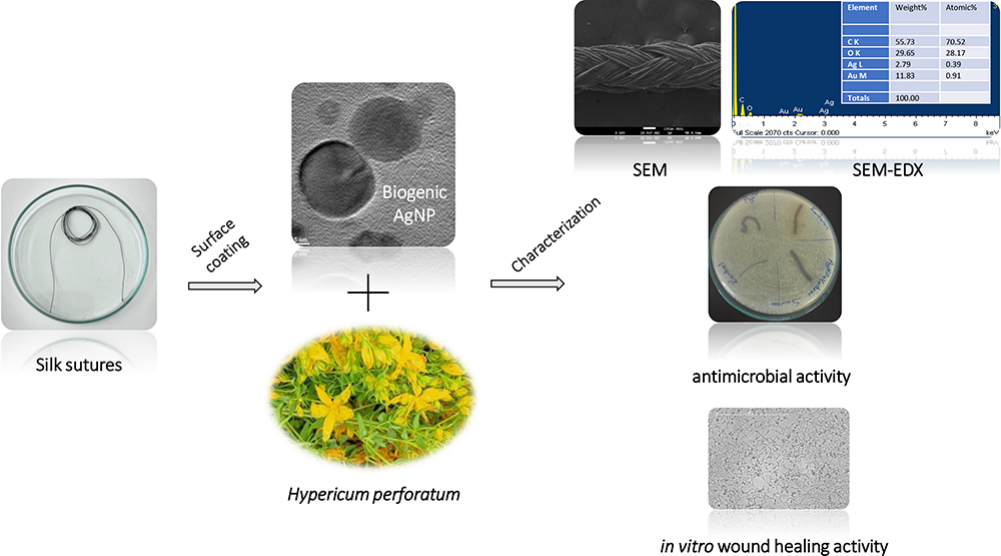

Suture-associated infections on surgical sites are known to be related to the surface characteristics of the sutures. The present study aimed to fabricate a novel functional suture for surgical procedures and characterize its antioxidative, antimicrobial, and in vitro wound healing properties. St John’s wort, *Hypericum perforatum*, extract (eHp), and biogenic silver nanoparticles (AgNPs) have been combined and used for coating the silk sutures. Antioxidant, antimicrobial capacity, and in vitro wound healing potential of the coated sutures have been examined. The morphological and microanalytical examination of the coated sutures was also performed by scanning electron microscopy (SEM) and energy-dispersive X-ray spectroscopy (EDS). According to the antioxidant activity tests, free radical scavenging and β-carotene linoleic acid tests revealed that the antioxidative potential of *H. perforatum* extract–AgNP combination (eHp–AgNP) at 10 mg/mL concentration was higher than those of positive controls, ascorbic acid and α-tocopherol. Coating the sutures with eHp-AgNP resulted in a remarkable inhibition activity of the sutures against *Staphylococcus aureus*, which is a pathogenic member of human microbiota. When compared with the control groups, it was investigated that coating the sutures with eHp–AgNP stimulated the cell migration of the fibroblasts to heal the artificial wound. Due to their beneficial effects, the eHp–AgNP-coated silk sutures might be a potential antibacterial and wound healing accelerator for surgical approaches.

## 1 Introduction

Surgical site infection (SSI) is a common postoperative infection that is likely to occur within 1 year after the surgery.^1^ Wound healing, which is a very complex biological process, can be affected by many suture-related factors, such as suture type and suturing technique. Sutures create a basis for surgical wound contamination which results in the accumulation of microorganisms and so causes the transfer of microorganisms to tissues. Sutures must therefore prevent or limit microbial adhesion and proliferation to those parts exposed to body fluids, thereby avoiding contamination inside the wound.^2^ Postoperative antibiotic treatment is widely used to prevent the wound from infection. To minimize the risks, antimicrobial suture coating ideas have been gaining much importance.^3^

Silk has been used as a suture material for decades and preferred by surgeons due to its excellent physical and handling properties.^4^ However, silk sutures tend to cause intense inflammation in the contact of the suture with tissue. The braided nature of silk sutures promotes bacterial accumulation, resulting in infections and damage to the local tissue.^5^ In order to minimize these problems, researchers have been focusing on the development of silk sutures with antimicrobial coatings that accelerate wound healing.

St John’s wort, *Hypericum perforatum*, is known as a medicinal plant which has been used in popular medicine and phytotherapy for its well-documented antiseptic effects.^6^ It has been traditionally used as a folk remedy for the management of wounds, bruises, skin ulcers, cuts, burns, contusions, depression, and myalgia.^7^

There are several antiseptic-coated sutures, such as triclosan and chlorhexidine, that have been commercially available for medical or veterinary use. However, the widespread use of triclosan for medicinal or cosmetic use is supposed to cause the development of bacterial resistance to this antiseptic.^8^ Therefore, inventing new antimicrobial agents that are highly active against microbial species is the main focus of many researches. Besides the synthetic chemicals, natural compounds including plant extracts are also used to coat the sutures for enhancing their antimicrobial features. Chitosan,^9^ aloe vera,^10^ and eugenol^11^ are some of the agents that have been applied onto the sutures.

Over the past decade, silver nanoparticles (AgNPs) have been the most widely investigated metallic nanoparticles due to their broad-spectrum antimicrobial properties and robust antimicrobial effectiveness against various bacteria, viruses, and fungi.^12^ Antimicrobial features of those particles have resulted in an increasing demand for their biomedical as well as industrial applications. Some of these products that are commercially available include biomedical devices, bone prostheses, contraceptive devices, surgical instruments, and wound dressings.^13^

Nowadays, nanotechnology has attracted considerable attention for fabricating antimicrobial sutures.^14^ Baygar et al.^13^ (2019) studied the in vitro antimicrobial characteristics and biocompatibility of silk sutures coated with biogenic AgNPs. In another report, Baygar^15^ (2020) reported that coating silk sutures with biogenic AgNPs and propolis resulted in enhanced antimicrobial and wound healing activities.

The synergistic antimicrobial effect of AgNPs and *Hypericum perforatum* extract with an enhanced healing capacity might be a novel approach to generate new suture materials. Within the present study, in vitro biological characterization of the newly designed sutures was evaluated in terms of antimicrobial activity and wound healing potential of the sutures. The morphology and elemental composition of coated sutures were assessed by scanning electron microscopy (SEM) and energy-dispersive X-ray spectroscopy (EDS).

## 2 Materials and Methods

### 2.1 Preparing the *H. perforatum* Extract Incorporated with Biogenic Silver Nanoparticles (eHp-AgNP)

The plant material was collected in the province of Mugla (Turkiye) in 2019 and dried at room temperature. Botanical identification was carried out, and a voucher specimen was deposited in the Department of Biology, Faculty of Sciences, Mugla Sıtkı Koçman University Mugla, Turkiye (Herbarium No: MUH 2796). The aerial parts of *H. perforatum* samples were extracted by a Soxhlet apparatus using ethanol (96%) as a solvent. The extraction process was continued until the colorless solution was obtained approximately at 3 h. The ethanol content of the solution was evaporated, and crude extract was stored in dark glass bottles at +4 °C.

The silver nanoparticles (AgNPs) used in this study were obtained via a biological route which is known as green synthesis. For this purpose, extracellular AgNP biosynthesis was performed by using *Streptomyces griseorubens* AU2 which is a Gram-positive aerobic bacteria isolated from soil.^16^

#### 2.1.1 Antioxidant Activity of eHp-AgNP

For investigating the antioxidative potential of the sutures, the antioxidant activity of the eHp-AgNP at 1, 5, and 10 mg/mL concentrations was determined using complementary test systems: DPPH (2,2-diphenyl-1-picryl-hydrazyl-hydrate) free radical scavenging activity and β-carotene-linoleic acid assay.^17,18^ Ascorbic acid and α-tocopherol at 1, 5, and 10 mg/mL concentrations were used as controls for both analyses.

### 2.2 Coating the Sutures with eHp-AgNP

Nonabsorbable USP size 3.0 plain braided silk sutures obtained from a local company (Dogsan, Istanbul, Turkey) were used as the base suture material for designing the sutures. Dip coating method was used to apply eHp-AgNP on the silk.^19,20^ For the coating process, a stock solution of eHp-AgNP (10%) containing 20 μg/mL AgNP (w/v) and 200 μg/mL eHp (w/v) was prepared with ethanol.

After the coating process, the coated samples were dried on glass plates at ambient temperature in a humid atmosphere and maintained for further analysis. Noncoated silk sutures, only *H. perforatum* extract (eHp)-coated sutures, and only biogenic AgNP-coated groups were used as controls.

### 2.3 Characterization of the Sutures Coated with eHp-AgNP

#### 2.3.1 Antimicrobial Activity

The antimicrobial activity of the sutures was determined against pathogenic strains *Staphylococcus aureus* ATCC 25923, *Enterococcus faecalis* ATCC 29212, *Candida albicans* ATCC 10231, and *Streptococcus mutans* ATCC 25575, using the standard agar plate method.^21^ Experiments were performed in triplicate, and the mean values ± standard deviation (SD) of the tests were calculated.

#### 2.3.2 In Vitro Wound Healing Potential

The wound healing efficiency of the sutures was assessed by in vitro wound healing scratch assay.^22^ Depending on the standard protocols reported by ISO 10993-5, suture fragments (1 cm length/mL) were immersed in fresh cell culture medium at 37 °C for 5 days to obtain the extracts.^23^ After 5 days of incubation, the extract products were collected. NIH-3T3 murine fibroblast cells were treated with the extract of the sutures, and the control group was prepared as the cells cultured in the basal medium. Representative images from each cell culture dish of the scratched areas were photographed using a Leica DM IL microscope (Leica Microsystems, Wetzlar, Germany) to estimate the relative migration of the cells.^15^

#### 2.3.3 Morphological and Microanalytical Characterization of the Sutures

The surface morphology of the eHp-AgNP-coated sutures was evaluated using SEM (JSM 7600F, JEOL, Japan), and the elemental composition was determined by EDS (Oxford Instruments, UK) combined with SEM. Sutures were sputter-coated with conductive gold for greater visualization (K550×, Emitech, UK). SEM and EDS measurements were performed in a high vacuum mode with a secondary electron detector using 15 kV for the accelerating voltage and 8 mm for the working distance.

## 3 Results

### 3.1 Morphological and Microanalytical Characterization of the Coated Sutures

**Figure 1:**
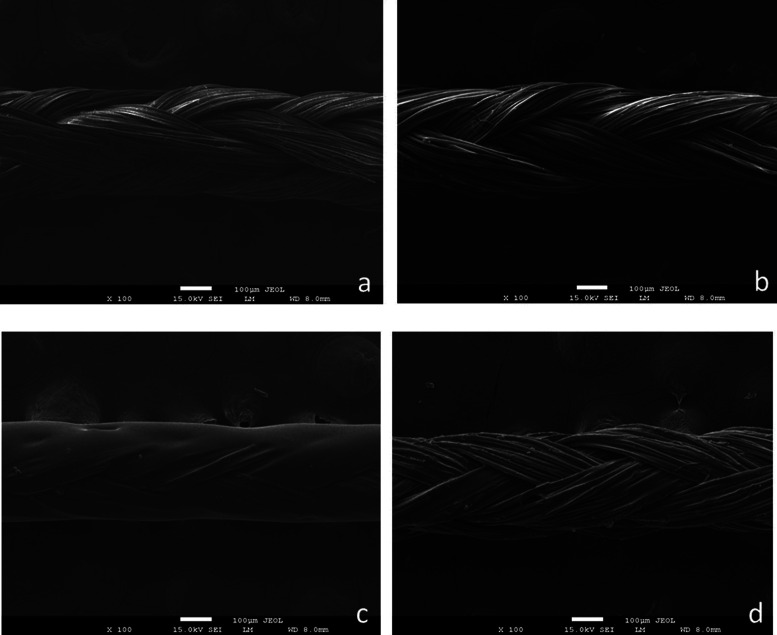
SEM micrographs of the noncoated (a), biogenic AgNP-coated (b), eHp-coated (c), and eHp-AgNP-coated (d) sutures (the bar represents 100 μm).

The SEM micrographs of the noncoated, biogenic AgNP-coated, eHp-coated, and eHp-AgNP-coated sutures and the uncoated suture (Control) are shown in Figure 1.

**Figure 2:**
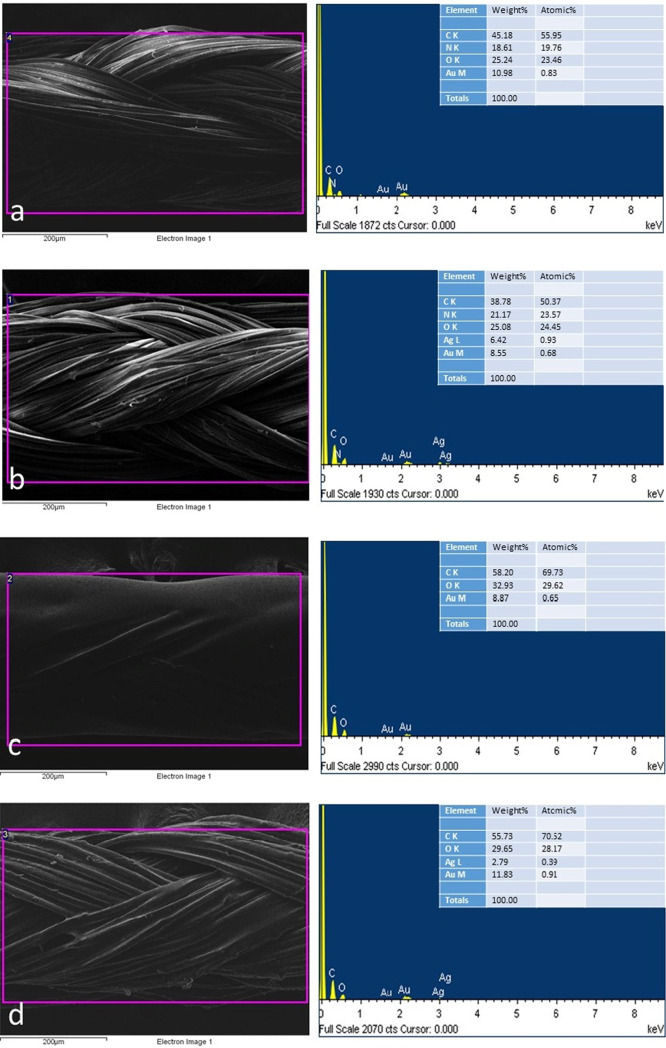
Energy-dispersive X-ray spectroscopy analysis of the noncoated (a), biogenic AgNP-coated (b), eHp-coated (c), and eHp-AgNP-coated (d) sutures. The image on the left side of the spectrum represents the selected area of the SEM image.

To examine the microanalytical composition of the coated sutures and the control groups, energy-dispersive X-ray spectroscopy (EDS) analysis was performed (Figure 2).

The spectrum of the energy-dispersive X-ray spectroscopy (EDS) analysis clarified the presence of silver (Ag) in the eHp-AgNP-coated suture. The major components of silk, carbon (C), nitrogen (N), and oxygen (O) were also found in the noncoated and AgNP-coated sutures. There were only C and O peaks in the spectra of the HP-coated suture, which might be due to the chemical composition of the *H. perforatum* extract. The gold (Au) peaks indicated the presence of gold elements due to the sputter-coating process applied for the SEM observation.

### 3.2 Antioxidant Activity of eHp-AgNP

The antioxidant activity test results of eHp-AgNP are given in Table 1. In the DPPH test system, the free radical scavenging activity of eHp-AgNP at 10 mg/mL concentration (94.81 ± 0.93%) was higher than those of positive controls, ascorbic acid (85.52 ± 0.69%) and α-tocopherol (81.18 ± 0.88%). The IC_50_ value of eHp-AgNP, which refers to the lowest concentration of the antioxidant necessary for 50% of the reactivity, was calculated as 3.07 mg/mL.

**Table 1:** Antioxidant Activity of eHp-AgNP

	concentration^a^ (mg/mL)			IC_50_ for free radical scavenging (mg/mL)	concentration^a^ (mg/mL)		
	10	5	1		10	5	1
	scavenging effect (%)				β-carotene/linoleic acid assay (%)		
*eHp-AgNP*	94.81 ± 0.93	69.76 ± 1.54	28.80 ± 0.17	3.07	97.94 ± 3.00	97.88 ± 1.35	89.07 ± 1.04
ascorbic acid	n.a.^b^	n.a.	85.52 ± 0.69	n.a.	n.a.	n.a.	92.43 ± 1.62
α- tocopherol	n.a.	81.18 ± 0.88	n.a.	n.a.	n.a.	84.41 ± 2.13	n.a.

a Test concentrations have been prepared as dilutions of the stock solution.

b n.a.: not available.

In the β-carotene-linoleic acid assay system, the method is based on the ability of the test solutions to decrease the oxidative losses of β-carotene in a β-carotene/linoleic acid emulsion.^24^ Similar to the DPPH test, the extract had the highest inhibition value for the co-oxidation reactions of linoleic acid and β-carotene at 10 mg/mL concentration, with 97.94 ± 3.00%.

### 3.3 Antimicrobial Activity of the Coated Sutures

**Figure 3:**
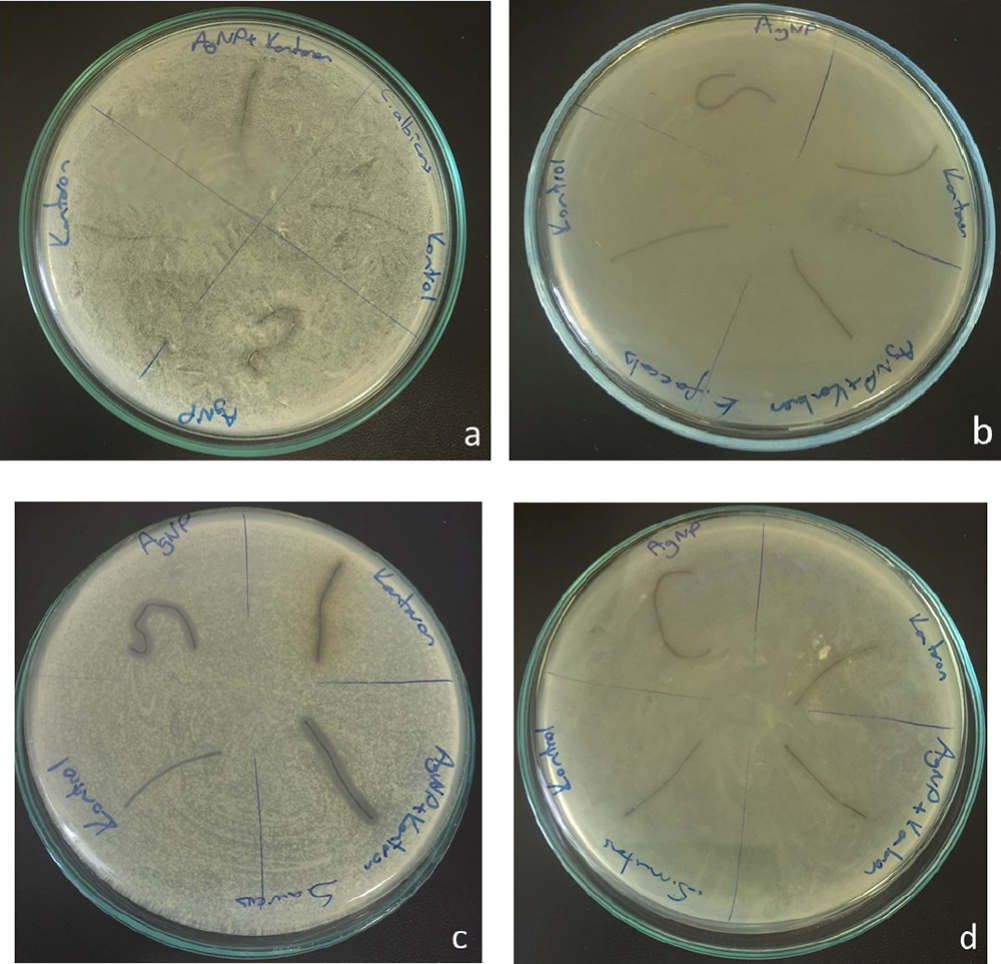
Images of the agar diffusion test for the antimicrobial activity of coated sutures. (a) *C. albicans*, (b) *E. faecalis*, (c) *S. aureus*, and (d) *S. mutans*.

According to the antimicrobial screening results, all coated sutures were found to be effective against *S. aureus*, while eHp-AgNP-coated sutures had a relatively higher inhibition zone (Figure 3). AgNP-coated and eHp-AgNP-coated sutures had inhibition activity against *C. albicans* but did not inhibit fungal growth completely. Similarly, the AgNP-coated suture had less antibacterial activity against *S. mutans*, whereas the eHp-AgNP-coated suture had partial inhibition activity.

### 3.4 In Vitro Wound Healing Potential of the Sutures

**Figure 4:**
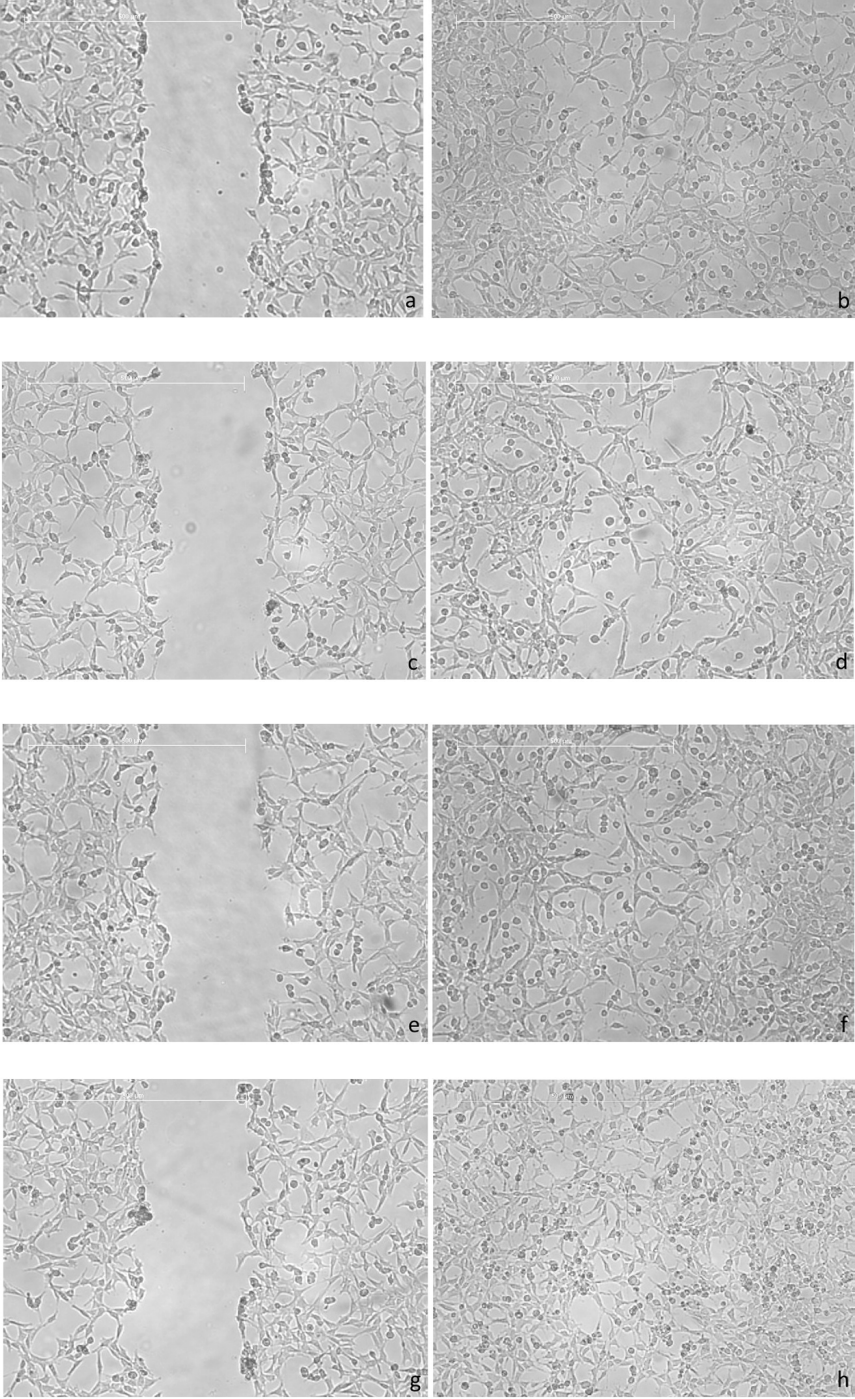
Representative images of the in vitro wound healing potential of the noncoated (a, b), eHp-coated (c, d), biogenic AgNP-coated (e, f), and eHp-AgNP-coated (g, h) sutures at time 0 (left side) and 24 h (right side) after incubation, respectively. The bar represents 500 μm.

The representative images indicated that the artificial scar of the noncoated group, which had been supposed to be the control, was closed after 24 h of the experiment (Figure 4). The migration of the fibroblasts was similar to that in the control group for eHp-coated sutures. For biogenic AgNP-coated sutures, there was a relative increase in the number of fibroblast cells at the wound closure area. When compared to the other groups, the migration of the fibroblasts to the scar was faster, and the wound closure outcomes were better for the eHp-AgNP-coated sutures. It can be inferred that the eHp-AgNP coating stimulated the cell migration after 24 h of the scar formation.

## 4 Discussion

Clinical and experimental data show that wound infections mostly begin around the sutured area, and even the severity of the infection in the sutureless area is less than that in sutured wounds.^25^ Sutures provide a conducive surface for bacterial adherence, colonization, and biofilm formation.^26^ Inhibition of the bacterial growth and a decrease in infection rate have been reported in sutures coated with antibacterial agents.^27^

Food and Drug Administration (FDA) approved the use of antimicrobial triclosan-coated absorbable suture in 2002.^28^ Triclosan that has a broad-spectrum inhibition activity against bacterial species is widely used in a variety of consumer products like toothpastes, soaps, hair shampoos, and facial cleansers.^29^ As a result of frequent use, it was reported that bacteria become resistant to triclosan via the target site modification mechanism.^30^ Resistance developed by pathogenic microorganisms has been thought as a serious concern for the use of triclosan.^31^ The excessive use of antibiotics has been known as the main reason for the existence of antibiotic-resistant bacteria in clinical facilities all around the world. Antimicrobial agents such as antiseptics, antibiotics, nanoparticles, or biotechnological products may be incorporated on sutures by using different approaches including dip-coating, surface modification and blending, and compounding.^32^ Natural compounds, such as plant extracts, have been potentially used for coating. Studies are recently focusing on the characterization of surgical sutures coated with natural or synthetic antimicrobial agents that have been designed as an alternative to coating the sutures with triclosan to provide antimicrobial properties.^28,32,33^

Within this study, a biocompatible and antimicrobial coating has been fabricated and applied onto the surgical sutures. The coating material has been characterized for its antioxidative potential before the coating process. The wound healing function of the *H. perforatum* extract and the antimicrobial function of the biogenic AgNPs have been combined in a novel suture coating material. At this purpose, characterizations, antimicrobial activity analysis, and in vitro wound healing tests have been performed on the sutures. It was inferred that the antimicrobial and wound healing activities of the *H. perforatum*-biogenic AgNP-coated sutures have been enhanced by the synergistic effect of the *H. perforatum* extract and biogenic AgNPs. The inhibition zones obtained by the agar diffusion method revealed that eHp-AgNP-coated sutures have inhibition zones higher than noncoated sutures. According to standard SNV 195920-1992, if the inhibition zone measurement of a material is higher than 1 mm, it is considered to have antimicrobial activity.^34^ In the present study, the highest inhibition zone was relatively observed against *S. aureus*, which is known to be the most common microorganism isolated from the wound infections.^35^

Nanotechnology applications open a new perspective for the fabrication of highly antimicrobial sutures. Researchers suggest that the antimicrobial effects of biologically synthesized metal nanoparticles are revealed by (a) causing an increase in the production of reactive oxygen species (ROS); (b) inactivating the functional enzymes in the respiratory chain by damaging the plasma membranes of microbial cells; (c) causing accumulation of metal ions in microbial membranes; (d) electrostatic attraction between nanoparticles and the cell; and (e) inhibiting of microbial proteins/enzymes through excess hydrogen peroxide (H_2_O_2_) production.^36^ The biogenic AgNPs used within the present study have been intensively characterized for their antioxidant,^16^ antimicrobial, antibiofilm,^37^ mutagenic, antimutagenic,^38^ cytotoxic, and wound healing^39^ activities. The biosynthesized AgNPs were used for coating the silk sutures, and it was concluded that biogenic AgNP-coated sutures have enhanced biological activities when compared to the bare silk sutures.^13^ Within this study, it was aimed to enhance the bioactive features of the sutures by combining the biocompatible characteristics of *H. perforatum* with biogenic AgNPs. There are similar studies about silver nanoparticle-coated sutures in the literature. Syukri et al.^40^ coated nylon monofilament surgical sutures with biogenic silver nanoparticles and inferred that coated sutures exhibited a high bactericidal activity on wound pathogens (*S. aureus*, *Escherichia coli*, *Pseudomonas aeruginosa*, *Acinetobacter baumannii*, and *Klebsiella pneumoniae*) compared to the uncoated specimen. Guadarrama-Reyes et al.^41^ decorated the cat gut sutures with biologically synthesized AgNPs using *Heterotheca inuloides* and evaluated their antibacterial activity against *E. coli* and *S. aureus*. They suggested that this suture might be used alternatively to help reduce the excess antibiotic use. Similar to the present study, Gallo et al.^25^ coated the absorbable multifilament polyglactin 910 PGLA sutures with nanosilver and inferred that the presence of silver promoted the migration and proliferation of 3T3 murine fibroblasts.

As far as our knowledge, there is no study about coating the sutures with *H. perforatum* extracts. In the present study, surgical sutures coated with *H. perforatum* extract and *H. perforatum* extract–biogenic AgNP mixture showed better wound healing capacity than the noncoated sutures.

## 5 Conclusions

Surgical sutures cause SSI by creating a suitable surface environment for the attachment and proliferation of microorganisms. Among the current methods used in the fight against SSI is the use of antibiotic-coated sutures. However, the use of these antibiotics is losing their effectiveness due to increasing antibiotic resistance. Among the new generation of applications, the use of metallic nanoparticles stands out.

In this study, an antimicrobial suture with dual function of healing the wound as well as providing microbial adhesion has been developed. New antimicrobial and wound healer suture materials have been designed and intensively characterized. Combining the *H. perforatum* extract, a common traditional wound healer plant, and green-synthesized silver nanoparticles enhanced the biological activities of the silk sutures. It was observed that coating the suture material enhanced the antibacterial characteristic of the suture against *S. aureus*, which is a common pathogenic bacteria related to wound infections. In vitro wound healing assay revealed out that coated sutures were found to have higher wound healing potential when compared to the noncoated silk sutures, The newly designed sutures may be good alternatives to antibiotic-coated surgical sutures with their accelerated healing potential of the wounds and their protection capacity of the surgical wounds from microbial attacks.
